# Comparison of Housing First and Traditional Homeless Service Users in Eight European Countries: Protocol for a Mixed Methods, Multi-Site Study

**DOI:** 10.2196/14584

**Published:** 2020-02-05

**Authors:** Ronni Michelle Greenwood, Rachel M Manning, Branagh R O'Shaughnessy, Oisin Cross, Maria J Vargas-Moniz, Pascal Auquier, Massimo Santinello, Judith R Wolf, Anna Bokszczanin, Roberto Bernad, Håkan Källmén, Frederik Spinnewijn, José Ornelas, HOME_EU Consortium

**Affiliations:** 1 Psychology Department University of Limerick Limerick Ireland; 2 Applied Psychology Research Center Capabilities and Inclusion Instituto Superior de Psicologia Aplicada Instituto Universitário Lisbon Portugal; 3 Centre d'Études et de Recherche sur les Services de Santé et la Qualité de Vie La Timone Medical Campus, School of Medicine Aix-Marseille University Marseille France; 4 Department of Developmental and Social Psychology University of Padova Padova Italy; 5 Impuls - Netherlands Center for Social Care Research Radboud Institute for Health Science Nijmegen Netherlands; 6 Institute of Psychology Opole University Opole Poland; 7 Red de Apoyo a la Integración Sociolaboral Fundacion Madrid Spain; 8 Stockholm Prevents Alcohol and Drug Problems Stockholm Center for Psychiatry Research and Education Karolinska Institute Stockholm Sweden; 9 European Federation of National Organisations Working with the Homeless Brussels Belgium; 10 see Authors' Contributions

**Keywords:** homeless services, Housing First, recovery, capabilities

## Abstract

**Background:**

Homeless services expend considerable resources to provide for service users’ most basic needs, such as food and shelter, but their track record for ending homelessness is disappointing. An alternative model, Housing First, reversed the order of services so that homeless individuals are offered immediate access to independent housing, with wraparound supports but no treatment or abstinence requirements. Although the evidence base for Housing First’s effectiveness in ending homelessness is robust, less is known about its effectiveness in promoting recovery.

**Objective:**

The objective of this research is to compare rehabilitation- and recovery-related outcomes of homeless services users who are engaged in either Housing First or traditional staircase services in eight European countries: France, Ireland, Italy, the Netherlands, Poland, Portugal, Spain, and Sweden.

**Methods:**

A mixed methods, multi-site investigation of Housing First and traditional services will compare quantitative outcomes at two time points. Key rehabilitation outcomes include stable housing and psychiatric symptoms. Key growth outcomes include community integration and acquired capabilities. Semistructured interviews will be used to examine service users’ experiences of environmental constraints and affordances on acquired capabilities to identify features of homeless services that enhance service users’ capabilities sets. Multi-level modelling will be used to test for group differences—Housing First versus traditional services—on key outcome variables. Thematic analysis will be used to understand the ways in which service users make sense of internal and external affordances and constraints on capabilities.

**Results:**

The study is registered with the European Commission (registration number: H2020-SC6-REVINEQUAL-2016/ GA726997). Two press releases, a research report to the funding body, two peer-reviewed articles, and an e-book chapter are planned for dissemination of the final results. The project was funded from September 2016 through September 2019. Expected results will be disseminated in 2019 and 2020.

**Conclusions:**

We will use the findings from this research to formulate recommendations for European social policy on the configuration of homeless services and the scaling up and scaling out of Housing First programs. From our findings, we will draw conclusions about the setting features that promote individuals’ exits from homelessness, rehabilitation, and recovery.

**International Registered Report Identifier (IRRID):**

RR1-10.2196/14584

## Introduction

### Background

The personal costs of homelessness are significant and multidimensional. Individuals who experience chronic homelessness are more likely to have mental health and/or substance misuse problems, experience victimization, and have fewer opportunities to develop positive identities or to participate in valued social activities than the general population [1-3]. European social policies that reverse homelessness rather than manage it have been quite limited and, as one consequence, homeless services expend considerable resources to provide for service users’ most basic needs, such as food and shelter. Increasingly, researchers have begun to direct their efforts toward understanding how to improve the structure of homeless services so they can do more to reverse homelessness and ameliorate its costs to individuals and society [4,5]. The aim of this study is to examine the relationship of homeless services settings to service users’ recovery experiences.

A growing body of evidence suggests that the traditional structure of homeless services, which follows a model of care that is variously referred to as “treatment first,” “continuum of care,” or “staircase,” limits homeless individuals’ potential for recovery in terms of both rehabilitation and growth [6]. A deficits model of homelessness, in which attributions for chronic homelessness focus on individuals’ mental illness, substance misuse, and poor decision making, underpins the structure of treatment-first programs [7]. Consequently, the setting features of traditional services encourage compliance through treatment and sobriety prerequisites, a range of house rules and regulations, and the promise of independent accommodation [8]. Failure to comply with setting rules can result in moves back down the continuum to more restrictive housing arrangements or even eviction [6,9].

The underwhelming track record of traditional services was highlighted and critiqued by both the consumer and recovery movements; both questioned the assumptions that underpin the treatment-first model and its setting features [10,11]. An alternative model of homeless services delivery that is based in principles of consumer choice, empowerment, and recovery was introduced in New York City in 1992 [12,13]. Originally designed to serve chronically homeless adults with serious mental illness who may also have a co-occurring substance use disorder, Housing First reversed the order of services to provide independent, scatter-site apartments with no treatment preconditions or abstinence requirements. Support services are tailored to clients’ preferences and needs and are provided 24/7 by either Assertive Community Treatment or Intensive Case Management teams. Housing First programs typically require service users to meet weekly with staff members, contribute 30% of their income toward the cost of rent, and comply with a standard lease agreement. Despite significant opposition to the model and skepticism from service providers and stakeholders [11,14], initial tests of Housing First returned impressive rates of stable housing [15,16] and cost-effectiveness [17,18] compared to traditional services. Critics’ fears about increased substance use and psychiatric symptoms have not been supported by research findings [14,19]. There is evidence that greater consumer choice afforded to individuals in Housing First programs fosters greater personal mastery, which, over time, predicts fewer psychiatric symptoms [20].

Since its first implementation, Housing First has been disseminated across the globe. The first randomized and controlled trial was conducted in New York City, in which Housing First was compared to treatment as usual [21,22]. The largest randomized trial of Housing First thus far was conducted in Canada [23] and a third randomized trial was conducted in France [24]. The positive outcomes of these trials spurred widespread dissemination and nonexperimental pilot and demonstration projects [4,5]. Although the exact number of Housing First programs is unknown, it has been widely disseminated in North America, Europe, Australia, and New Zealand [25,26]. Despite Housing First’s widespread reach and strong evidence base, most European homeless services continue to follow the treatment-first model [27]. Researchers and practitioners continue to work together to expand the evidence base for Housing First through rigorous experimental and observational trials. This study’s comparison of Housing First and traditional services in eight European countries aims to demonstrate not only that Housing First reverses homelessness in these different contexts, but to identify the setting features that explain how it works and, in doing so, produce translational findings that may have widespread influence in policy and practice.

### Homeless Service Features and Service Users’ Recovery Experiences

When homeless services providers, stakeholders, and policy makers advocate for new Housing First programs, or for the reconfiguration of traditional treatment-first programs toward *housing-led* services, they tend to be motivated by ambitions to develop programs that facilitate service users’ exits from homelessness; they are also motivated to promote service users’ empowerment, personal growth, and freedom to participate in valued activities and roles [28,29]. Taken together, this goal can be conceptualized in terms of facilitating “a life worth living,” which is defined in the capabilities approach as an individual’s freedom to do and to be [30,31]. More specifically, capabilities are the opportunities that are realistically available to a person, which are determined by environmental and internal factors. Environmental factors can operate as either affordances or constraints on an individual’s ability to develop, maintain, or exercise internal capacities to freely enact desired behaviors. Internal affordances and constraints include, but are not limited to, a person’s intellectual abilities, physical and mental health, relationship to alcohol and illicit substances, skills, traits, motivations, and characteristic adaptations. For example, childhood poverty and neglect are environmental constraints that undermine an individual’s ability to obtain adequate education or develop the kinds of intra- and interpersonal skills required for a range of occupations, roles, and activities [32]. Functionings are those capabilities that a person freely chooses to enact.

Situations of extreme social exclusion such as homelessness are environmental constraints; that is, they are forms of inequality and unfairness that block an individual’s opportunities to develop new internal affordances or restore affordances that may have been lost. Indeed, homelessness has been described as capabilities failure [28] and capabilities deprivation [33]. Homeless services settings are important mediating structures that can broaden or constrict the environmental affordances on an individual’s freedom to do or to be [28,29]. Homeless services can also facilitate or constrain a person’s opportunities to develop the kinds of internal affordances, such as education, skills, psychiatric symptom management, or effective self-regulation of substance use, that are prerequisites to functionings in valued activities or socially valued identities and roles [28,29]. We aim to identify the setting features of homeless services (ie, environmental affordances and constraints) that affect service users’ abilities to develop useful skills and abilities (ie, internal affordances) that broaden their capabilities sets and enhance their central functionings.

Although a growing evidence base reports the effectiveness of Housing First interventions for reducing homelessness for adults with complex support needs [22,34], there is more to learn about the specific setting features that operate as mechanisms through which these results may be achieved. Three important setting features are choice over housing and services, housing quality, and satisfaction with services. Previous research demonstrated that participants who were engaged with Housing First services consistently reported greater choice, better housing quality, and more satisfaction with services [5,18,35,36]. Among other important outcomes, consumer choice predicts greater mastery, stable housing, fewer psychiatric symptoms, and less problem-related substance use [15,16,35,37]. Perceived housing quality and service users’ satisfaction with their input into the treatment and services they receive have both been associated with positive recovery-related outcomes, including engagement with supports [38] and reduced substance use [39].

### This Study

In this paper, we describe the protocol for the Service Users’ Experiences Study, one prong of a larger project called *Homelessness as Unfairness*, or HOME_EU, which takes an ecological approach to understanding long-term homelessness in Europe. Citizens’ attitudes, social policy, service providers’ experiences, and service users’ experiences are each investigated in separate empirical studies. Our aim is to combine findings from the four studies into a body of translational knowledge that can be used to enhance European social policies so that countries may move beyond managing homelessness toward ending it.

In the Service Users’ Experiences Study, we aim to investigate the features of homeless services hypothesized to function as key environmental affordances and constraints on service users’ recovery and capabilities. We aim to understand how persistent homelessness thwarts individuals’ basic liberties and equality aspirations. The capabilities approach [40] provides the theory-driven framework that guides our work on this project, which we will use to interpret our findings and propose practical guidelines to promote social justice in homeless policy and homeless services settings. The HOME_EU Consortium will combine findings from the Service Users’ Experiences Study with findings from our studies with citizens, policy makers, and service providers. This will produce a translational multidimensional conceptualization of homelessness across eight European countries that will inform social policy at the European and national levels and encourage best practices in homeless services that promote recovery and inclusion in civil society.

### Research Design

Our study uses a mixed methods design and is being conducted in eight European countries: France, Ireland, Italy, Poland, Portugal, the Netherlands, Spain, and Sweden. Our design is correlational and not randomized. We will recruit participants who are already engaged with either Housing First or traditional services and collect quantitative data via questionnaires at two time points: baseline (T0) and follow-up (T1). This will allow us to control for baseline nonequivalence on demographic characteristics and other individual differences, such as lifetime homelessness, length of time in current accommodation, alcohol and substance use, education, and physical health. A subset of participants from each country who completed the questionnaires will complete an in-depth, qualitative, capabilities interview. In this paper, we describe the core procedures and methods that will be completed across all eight study sites.

### Objectives

The first objective of this study is to compare Housing First and traditional services regarding key service and support features hypothesized to promote recovery in terms of both rehabilitation and growth. The second objective is to explore participants’ subjective experiences of environmental and internal affordances and constraints on their functionings and capabilities, especially in the areas of valued social roles, activities, relationships, and responsibilities [28,30,31,41,42].

The specific objectives of the Service Users’ Experiences Study are as follows:

1. Determine whether Housing First and traditional services are differentiated on key setting features previously demonstrated to be linked to recovery indicators.
2. Determine whether implementations of Housing First across eight different European contexts are consistent with one another and consistently differentiated from traditional services on these key setting features.
3. Determine whether setting features predict recovery outcomes at the second time point (T1), controlling for baseline (T0) scores.
4. Understand service users’ subjective experiences of their own recovery of human rights, defined as capabilities, and the ways in which these experiences are afforded or constrained by their engagement with Housing First or traditional homeless services.

## Methods

### Quantitative Methods and Analysis

#### Recruitment and Data Collection

##### Overview

Consortium partners in each country will use their existing links to Housing First programs and traditional homeless services to recruit participants to the study. Partners in France, Ireland, Italy, Portugal, and Spain have been directly involved in Housing First pilot and demonstration projects, so they may directly contact participants to invite them to participate in this research. Researchers will liaise with key workers, team leaders, and program managers employed in either Housing First programs or traditional services for assistance with recruiting additional participants to the study so that we can achieve our target sample size.

##### Questionnaire Administration

Research interviewers will meet individually in a quiet location chosen by participants. After a short ice-breaker conversation intended to build rapport, the researcher will explain the study and obtain informed consent before administering the questionnaire. The 13 measures included in the baseline questionnaire are presented in Table 1. Research interviewers will read each item to participants using a standardized procedure and record participants’ responses on the questionnaires. When the questionnaire is complete, the researcher will ask permission to contact the person to complete the questionnaire 6 months later.

##### Data Management

Participants’ responses will be entered into a standardized SPSS, version 24.0 (IBM Corp), data file template that will be used in every site to ensure equivalence of data entry. Several steps will be, or have been, taken to ensure data quality. First, we will administer measures previously used with this population to maximize measure validity and reliability. Second, interviewers will follow a data entry protocol and receive ongoing support via telephone, email, and face-to-face meetings. Third, the type and range of data values and mandatory entry were built into entry fields in the database. Fourth, questions from interviewers were fielded centrally and decision rules were made where necessary and circulated to all partners. Fifth, the authority to change data elements will be restricted to a small team [43].

#### Sample Size and Retention Plan

Our target sample for the second time point (T1) is 480 participants (Housing First, n=240, and traditional services, n=240). Because we anticipate attrition between T0 and T1, we intend to oversample each group at baseline. Based on prior research [22,36], we anticipate greater attrition in the traditional services group, so we aim to sample 38 Housing First participants and 45 traditional service participants in each of the eight countries at T0 (N=664). In order to maximize follow-up, we have adapted Stefancic and colleagues’ guidelines [44]. Specifically, at the baseline data collection meeting, researchers will ask participants to predict where they will be living 6 months later; to provide a range of contacts for family members, friends, and service providers; and to contact the research team if they change their phone number or move to a new residence. Participants will be compensated with a €20 shopping voucher for each questionnaire and interview they complete.

#### Participants

To be eligible to participate in this study, potential participants must meet the following inclusion criteria: be 18 years of age or older to legally consent to participate; have spent 6 or more months homeless in their lifetime; be currently engaged with homeless services, either Housing First or traditional services; and be sufficiently proficient in the language of the country in which they reside to understand all the questionnaire items. Exclusion criteria include the following: unable to provide consent at time of data collection because of active psychosis or inebriation, less than 18 years of age, have insufficient proficiency in the questionnaire language, have spent fewer than 6 months homeless in their lifetime, and not currently engaged with a homeless service.

#### Measures

##### Overview

The measures selected for this study are listed in Table 1 [36,45-57]. There are three main categories of measures: setting and support features, rehabilitation-related recovery, and growth-related recovery. Although some measures have already been translated into some of the languages represented in this study, most measures had to be translated into most languages. The Consortium agreed to adopt standard translation-back translation procedures for cross-cultural research [58]. Questions or disagreements about translation were discussed among Consortium partners until they reached consensus.

**Table 1:** Quantitative measures used for baseline (T0) and follow-up (T1) data collection.

Domain,Variables		Instruments
**Setting and support features**		
	Working alliance	Working Alliance Inventory—Participant (Horvath and Greenberg, 1989) [47]
	Service satisfaction	Self-Help Agency Satisfaction Scale (Segal et al, 2000) [46]
	Housing quality	Choice in Housing and Services (Srebnik et al, 1995) [45]
	Choice	Perceived Housing Quality and Choice/Control (Toro et al, 1997) [36]
**Rehabilitation-related recovery**		
	Housing status	European Typology on Homelessness and Housing Exclusion (FEANTSA^a^, 2005) [52]
	Psychiatric symptoms	Colorado Symptom Index (Shern et al, 1994) [48]
	Alcohol and drug use	Alcohol Use Disorders Identification Test (Babor et al, 2001) [49] Drug Use Disorders Identification Tool (Berman et al, 2005) [50]
	Physical health	General Self-Rated Health (DeSalvo et al, 2006) [51]
**Growth-related recovery**		
	Mastery	Mastery Scale (Pearlin and Schooler, 1978) [53]
	Capabilities	Capabilities Scale
	Recovery	Recovery Assessment Scale (Giffort et al, 1995) [54]
	Community integration	Community Integration Measure (Aubry and Myner, 1996; Segal and Aviram, 1978) [55,56]
	Distal social support	Distal Social Support Scale (Wieland et al, 2007) [57]

^a^FEANTSA: European Federation of National Organisations Working with the Homeless.

##### Setting and Support Features

Based on previous research on homeless services and theories of recovery, we identified four dimensions of service and support features to measure. These are subjective measures of service users’ perceptions of the setting that assess choice over housing and services [45], housing quality [36], satisfaction with involvement with services [46], and working alliances with service providers [47].

Objective setting features were identified from the literature on Housing First and staircase services, as well as our own experiences of these environments. These include the following service setting features: congregate or scatter-site housing, private or shared bedroom, private or shared bathroom, mixed or segregated gender, tolerate alcohol use on-site or not, tolerate drug use on-site or not, tolerate intoxication on-site or not, fixed length of stay or not, treatment required or not, set meal times or not, and curfews or not.

##### Rehabilitation-Related Recovery

For homeless adults, rehabilitation-related recovery is often conceptualized as successfully maintaining independent accommodation, decreased psychiatric symptom frequency, decreased problem-related alcohol and drug use, and improved physical health. We selected measures with established validity and reliability to measure these rehabilitation-related recovery indicators, which are listed in Table 1. Specifically, we included the Colorado Symptom Index [48] as well as measures of alcohol and drug use [49,50] and physical heath [51]. We also created a measure of housing status based on the European Typology on Homelessness and Housing Exclusion [52].

##### Growth-Related Recovery

Growth-related recovery in homelessness is also multidimensional, so we included a range of measures to capture the dimensions that are commonly the focus of key stakeholders’ attention. We included intrapersonal measures, such as mastery [53], hope, meaning of life, quality of life, and empowerment [54]. We also included interpersonal measures, such as community integration [55,56] and distal social support [57]. A measure of capabilities was developed and its content was validated for use in the Service Users’ Experiences Study. It consists of 54 items adapted from the capabilities framework [42] and the Acquired Capabilities Questionnaire for Community Mental Health [59].

#### Statistical Analysis

We plan to compare participants who are engaged in Housing First programs to participants engaged in traditional services to determine whether they differ on service and support features, rehabilitation-related recovery outcomes, and growth-related recovery outcomes at baseline and at follow-up. Based on previous experience [60] in which participants chose to skip items and even entire measures, we expect to have missing data on most measures. We also expect attrition at T1 because participants have died, cannot be located, or do not wish to complete the questionnaire a second time. We will use Little’s *missing completely at random* test to determine whether data is *missing completely at random*, *missing at random*, or *neither*. We will manage data that is *missing completely at random* or *missing at random* using one of two approaches, depending on the research question, statistical technique, and software package. In the first technique, we will use expected maximization imputation techniques [61]. We will employ this technique with cross-sectional analyses of variance and multiple regression with SPSS, version 24.0 (IBM Corp). In the second technique, we will employ maximum likelihood in multi-level modelling with Mplus, version 8.2 (Muthén & Muthén) [62].

We will use T0 data to control for differences at baseline while examining patterns of association at T1. As individual data will be nested within program data, we will use multi-level modelling to account for Level 1 and Level 2 differences in intercepts, slopes, and intercept-slope covariances while controlling for baseline scores, country of residence, and demographic factors. If there are significant differences in the intercepts or slopes for recovery outcomes, we will determine whether these differences are accounted for by program membership—Housing First versus traditional services—and setting features.

### Qualitative Methods and Analysis

#### Participants

From the full sample of participants who complete the quantitative questionnaire at T1, we will invite 10 participants in each country (Housing First, n=5, and traditional services, n=5) to complete a semistructured interview focused on the 10 domains of capabilities identified by Nussbaum [42]. Partners will select participants with an aim for gender and age balance, where possible. Given the demands of a qualitative interview, HOME_EU Consortium partners will be asked to select participants who, from their previous research encounters, seemed capable of engaging with questions and enjoyed talking but also those who were able to focus and effectively articulate their thoughts.

Partners will only recruit participants to the qualitative study if they consented to be contacted again about follow-up research. These potential participants will be invited to participate in a qualitative interview about their experiences of homeless services, either over the phone or in person. Participants will be told that the interview will take about 1 hour and will be audio recorded, their data will be anonymized, and they will receive a €20 shopping voucher in return for participation. If they agree, partners will arrange a time and place to meet the participant.

#### Semistructured Capabilities Interviews

One of the key objectives of *Homelessness as Unfairness* is to explore the capabilities sets [28,42,63] of homeless services users in Europe and to identify the ecological factors that enable or block these capabilities sets (eg, Maton, 2008 [64]). To achieve this objective, our aim for the qualitative interviews is to deeply explore the capabilities sets of 10 homeless services users (Housing First, n=5, and traditional services, n=5) in each country. We developed an interview guide to explore homeless services users’ subjective accounts of their central functioning capabilities [42] (see Table 1 and Greenwood et al, 2013 [27]). In developing this interview guide, we followed Shinn’s [28] suggestions to examine these capabilities sets in terms of participants’ behaviors or activities that a person freely chooses to do or not to do. We also aim to explore their subjective understanding of internal and external affordances and constraints, which are intrapersonal factors and environmental factors that the person perceives to either facilitate or restrict the range of their realistically possible capabilities.

#### Sampling and Recruitment

##### Participant Selection

A total of 5 Housing First services-engaged participants and 5 traditional services-engaged participants who complete the T1 quantitative questionnaire will be recruited in each country to complete the qualitative interview. When choosing and recruiting these participants, we will aim for gender balance, where possible, and aim to have a range of ages represented in the sample. Because the capabilities interview addresses many abstract concepts, such as freedom and rights, ideal candidates for the qualitative interviews are participants who engaged well with the quantitative questionnaire, who are reflective on their lives, and are able to focus their attention and compellingly describe their experiences. Because we do not want the experience of completing the capabilities interview to influence participants’ responses on the quantitative measures, the T1 questionnaire will be completed prior to the interview.

##### Procedures

Interviews will be prearranged to occur at a quiet location chosen by the participant. Research interviewers should greet the participant and have a short ice-breaker chat, then explain the purpose of the interview and give an indication of how long the interview will last. After obtaining informed consent, the interviewer will begin the interview. With the participant’s consent, the interview will be digitally recorded.

Researchers will follow the semistructured interview guide (available upon request from the first author) in a way that adapts to each participant’s responses. The interview guide is structured so that the interviewer can take the interviewee through a discussion of the domains of capabilities identified by Nussbaum [42]. The prompts for each of the domains are constructed so that the interviewer can gain insight not only into the types of choices that are made in these areas, but also the breadth of choices and the affordances and constraints on choices in each domain. The main purpose is to understand the “range of realistic possibilities” [28], that is, the capabilities set that is available to each interviewee, along with the forces they experience as either facilitating or constraining their capabilities.

The interviewers will ask the interviewees to describe themselves in terms of each domain, their range of capabilities in each domain, the things they could do but do not want to do in each domain, the things they cannot do in that domain but would like to, and the factors they experience as blocking or facilitating their capabilities in each domain. Once all the domains have been covered, the interviewer will ask the participant to reflect on the conversation and see if they have anything they would like to add or clarify. Participants will be thanked for their time and provided with a €20 shopping voucher in return for their contribution. They will be invited to contact the research team with any follow-up questions or comments.

#### Qualitative Analyses

Each digitally recorded interview will be transcribed verbatim and anonymized. A deductive coding scheme based on the capabilities domains will be used by all Consortium partners to code each transcript (available upon request from the first author). Two independent coders will code each interview and then meet to agree on the codes. The coded excerpts will then be translated into English using the procedures agreed upon by Consortium partners [58]. The two independent coders will then agree on the English translation of the coded excerpts. The interview codes will be provided to the lead researchers on the Service Users’ Experiences Study, who will collate the codes obtained in each country and enter them into NVivo 11 software (QSR International) for thematic analysis [65].

### Data Synthesis

Qualitative and quantitative data from the Service Users’ Experiences Study component of the HOME_EU study of *Homelessness as Unfairness* will be triangulated with findings from our studies with citizens, policy makers, and service providers. Our aim is to produce a holistic understanding of homelessness across eight European countries that can be used to shape national and European social policy, encourage best practices in homeless services, reverse unfairness and inequality associated with homelessness, and promote recovery and inclusion in civil society.

### Ethics and Data Access

The *Homelessness as Unfairness* project received ethics approval from the lead partners’ (JO and MJVM) home university’s research ethics committee (ie, institutional review board). Each of the Consortium partners negotiated ethics approval with their home institutions. For example, the Irish team submitted evidence of approval from the lead partner’s university, along with a description of the work to be carried out with participants in Ireland.

A separate ethics work package was developed to ensure research integrity and protection of participants and researchers. Access to the data will be controlled by each Consortium partner. Completed questionnaires will be anonymized and stored separately from signed informed consent forms. Anonymized data will be input into SPSS, version 24.0 (IBM Corp), files stored on password-protected computers. Data files will be shared electronically via a secure data-sharing program. Access to the data will be limited to core personnel working on the project.

## Results

The study is registered with the European Commission (registration number: H2020-SC6-REVINEQUAL-2016/ GA726997). Two press releases, a research report to the funding body, two peer-reviewed articles, and an e-book chapter are planned for dissemination of the final results. The project was funded from September 2016 through September 2019. Expected results will be disseminated in 2019 and 2020.

## Discussion

We will use the findings from this research to formulate recommendations for European social policy on the configuration of homeless services and the scaling up and scaling out of Housing First programs. From our findings, we will draw conclusions about the setting features that promote individuals’ exits from homelessness, rehabilitation, and recovery.

## Appendix

Multimedia Appendix 1 Grant peer review and funding.

Multimedia Appendix 2 Proposal evaluation form and peer-reviewer comments from the European Commission.

## Abbreviations

FEANTSA: European Federation of National Organisations Working with the Homeless
T0: baseline, first time point
T1: follow-up, second time point
