# Supplementary material for: Comparison of Housing First and Traditional Homeless Service Users in Eight European Countries: Protocol for a Mixed Methods, Multi-Site Study
Source: JMIR Res Protoc. 2020 Feb 5;9(2):e14584. doi: 10.2196/14584 (PMC7055843; doi:10.2196/14584)
Supplement: Multimedia Appendix 1 [file resprot_v9i2e14584_app1.pdf]

HOME_EU

Project ID: 726997
Funded under:
H2020-EU.3.6.1.2. - Trusted organisations, practices, services and policies that are necessary to build resilient, inclusive, participatory, open and creative societies in Europe, in particular taking into account migration, integration and demographic change

Homelessness as unfairness

From 2016-10-01 **to** 2019-09-30, ongoing project

Project details

Total cost:	Topic(s):
EUR 2 111 992,50	REV-INEQUAL-01-2016 - An empirically informed European theory of justice and fairness
**EU contribution:**	**Call for proposal:**
EUR 2 111 992,50	H2020-SC6-REV-INEQUAL-2016 See other projects for this call
**Coordinated in:**	**Funding scheme:**
Portugal	RIA - Research and Innovation action

Objective

Empirically informing a European theory of justice is a complex and challenging endeavour, however the emergence of current social crisis, and the resulting inequalities and unfairness, bring about the need to revise the premises that facilitate translation of the theory into concrete guidance to effective social policies and coherent programs and practices.

To respond to this challenge, a trans-disciplinary Consortium has been organized to provide a comprehensive series of empirical data, in different ecological levels, in order to understand differences in perceptions of inequality.

Through a case study on an extreme expression of inequality and unfairness - LONG-TERM HOMELESSNESS – organized in a multi-method and convergent design, HOME_EU is focused on understanding: a) How much inequality do EU Citizens accept regarding Homelessness; b) How the people with a lived-experience of Homelessness (both present and past) perceive the opportunities, choices and capability gains with the services and the existing social policies; c) What strategies consider the service providers to be more effective in reversing Homelessness; d) How social policies and policy key stakeholders contribute to effectively reverse Homelessness; and e) Develop a generalizable indicator (correlating the different ecological levels of analysis) based on the data gathered by each partner country on the key elements of policy and program efficacy.

We believe that with this journey into an extreme situation, we are able to generate translational knowledge about the ecology of long-term Homelessness and contribute towards the advancement of an empirically based EU theory & practice of justice as fairness.

Coordinator

ISPA CRL
RUA JARDIM DO TABACO 34
1149 041 LISBOA
Portugal

Portugal

EU contribution: EUR 417 696,25

Activity type: Higher or Secondary Education Establishments

Participants

UNIVERSITY OF LIMERICK
NATIONAL TECHNOLOGICAL PARK, PLASSEY
- LIMERICK
Ireland

Ireland

EU contribution: EUR 264 493,75

Activity type: Higher or Secondary Education Establishments

ASSOCIACAO PARA O ESTUDO E INTEGRACAO PSICOSOCIAL
AV ANTONIO JOSE DE ALMEIDA 26
1000 043 LISBOA
Portugal

Portugal

EU contribution: EUR 25 851,25

Activity type: Other

UNIVERSITA DEGLI STUDI DI PADOVA
VIA 8 FEBBRAIO 2
35122 PADOVA
Italy

Italy

EU contribution: EUR 207 793,75

Activity type: Higher or Secondary Education Establishments

CRESCER NA MAIOR - ASSOCIACAO DE INTERVENCAO COMUNITARIA
AVENIDA DE DINIZ 49 2DT
2675 333 ODIVELAS
Portugal

Portugal

EU contribution: EUR 24 906,25

Activity type: Other

FEDERATION EUROPEENNE D'ASSOCIATIONS NATIONALES TRAVAILLANT AVEC LES SANS-ABRI AISBL
CHAUSSEE DE LOUVAIN 194
1210 BRUXELLES
Belgium

Belgium

EU contribution: EUR 22 968,75

Activity type: Other

FUNDACION RED DE APOYO A LA INTEGRACION SOCIOLABORAL RAIS
CALLE ARDEMANS 42
28028 MADRID
Spain

Spain

EU contribution: EUR 210 706,25

Activity type: Other

UNIWERSYTET OPOLSKI
PL MIKOLAJA KOPERNIKA 11A
45 040 OPOLE
Poland

Poland

EU contribution: EUR 38 437,50

Activity type: Higher or Secondary Education Establishments

STICHTING KATHOLIEKE UNIVERSITEIT | Netherlands
GEERT GROOTEPLEIN NOORD 9
6525 EZ NIJMEGEN
Netherlands

EU contribution: EUR 133 268,75

Activity type: Higher or Secondary Education Establishments

KAROLINSKA INSTITUTET | Sweden
Nobels Vag 5
17177 STOCKHOLM
Sweden

EU contribution: EUR 131 203,75

Activity type: Higher or Secondary Education Establishments

CAMARA MUNICIPAL DE LISBOA | Portugal
PACOS DO CONCELHO PRACA DO MUNICIPIO
1100-365 LISBOA
Portugal

EU contribution: EUR 26 641,25

Activity type: Public bodies (excluding Research Organisations and Secondary or Higher Education Establishments)

UNIVERSITE D'AIX MARSEILLE | France
Boulevard Charles Livon 58
13284 Marseille
France

EU contribution: EUR 608 025

Activity type: Higher or Secondary Education Establishments

Last updated on 2016-11-30
Retrieved on 2017-02-10

Permalink: http://cordis.europa.eu/project/rcn/205653_en.html
© European Union, 2017
