# Supplementary material for: Comparison of Housing First and Traditional Homeless Service Users in Eight European Countries: Protocol for a Mixed Methods, Multi-Site Study
Source: JMIR Res Protoc. 2020 Feb 5;9(2):e14584. doi: 10.2196/14584 (PMC7055843; doi:10.2196/14584)
Supplement: Multimedia Appendix 2 [file resprot_v9i2e14584_app2.pdf]

Associated with document Ref. Ares(2016)2346327 - 20/05/2016

Proposal Evaluation Form

EUROPEAN COMMISSION

Horizon 2020 - Research and Innovation Framework Programme

Evaluation Summary Report - Research and innovation actions/Innovation actions

**Call:**	H2020-SC6-REV-INEQUAL-2016
**Funding scheme:**	RIA
**Proposal number:**	726997
**Proposal acronym:**	HOME_EU
**Duration (months):**	36
**Proposal title:**	Homelessness as unfairness
**Activity:**	H2020-REV-INEQUAL-2016

N.	Proposer name	Country	Total Cost	%	Grant Requested	%
1	ISPA - INSTITUTO SUPERIOR DE PSICOLOGIA APLICADA, CRL	PT	420,571	19.89%	417,696	19.78%
2	UNIVERSITY OF LIMERICK	IE	264,494	12.51%	264,494	12.52%
3	AEIPS - Associação para o Estudo e Integração Psicossocial	PT	25,851	1.22%	25,851	1.22%
4	UNIVERSITA DEGLI STUDI DI PADOVA	IT	207,794	9.83%	207,794	9.84%
5	Crescer Na Maior - Associação de Intervenção Comunitária	PT	24,906	1.18%	24,906	1.18%
6	FEDERATION EUROPEENNE D'ASSOCIATIONS NATIONALES TRAVAILLANT AVEC LES SANS-ABRI AISBL	BE	22,969	1.09%	22,969	1.09%
7	Fundación Red de Apoyo a la Integración Sociolaboral - RAIS	ES	210,706	9.96%	210,706	9.98%
8	UNIWERSYTET OPOLSKI	PL	38,438	1.82%	38,438	1.82%
9	STICHTING KATHOLIEKE UNIVERSITEIT	NL	133,269	6.30%	133,269	6.31%
10	KAROLINSKA INSTITUTET	SE	131,204	6.20%	131,204	6.21%
11	CAMARA MUNICIPAL DE LISBOA	PT	26,641	1.26%	26,641	1.26%
12	UNIVERSITE D'AIX MARSEILLE	FR	608,025	28.75%	608,025	28.79%
	Total:		2,114,868		2,111,992	

Abstract:
Empirically informing a European theory of justice is a complex and challenging endeavour, however the emergence of current social crisis, and the resulting inequalities and unfairness, bring about the need to revise the premises that facilitate translation of the theory into concrete guidance to effective social policies and coherent programs and practices. To respond to this challenge, a trans-disciplinary Consortium has been organized to provide a comprehensive series of empirical data, in different ecological levels, in order to understand differences in perceptions of inequality. Through a case study on an extreme expression of inequality and unfairness - LONG-TERM HOMELESSNESS – organized in a multi-method and convergent design, HOME_EU is focused on understanding: a) How much inequality do EU Citizens accept regarding Homelessness; b) How the people with a lived-experience of Homelessness (both present and past) perceive the opportunities, choices and capability gains with the services and the existing social policies; c) What strategies consider the service providers to be more effective in reversing Homelessness; d) How social policies and policy key stakeholders contribute to effectively reverse Homelessness; and e) Develop a generalizable indicator (correlating the different ecological levels of analysis) based on the data gathered by each partner country on the key elements of policy and program efficacy. We believe that with this journey into an extreme situation, we are able to generate translational knowledge about the ecology of long-term Homelessness and contribute towards the advancement of an empirically based EU theory & practice of justice as fairness.

Evaluation Summary Report

Evaluation Result

Total score: 14.50 (Threshold: 10)

Form information

SCORING

Scores must be in the range 0-5.

Interpretation of the score:

*0– The **proposal fails to address the criterion** or cannot be assessed due to missing or incomplete information.*

*1– **Poor.** The criterion is inadequately addressed, or there are serious inherent weaknesses.*

*2– **Fair.** The proposal broadly addresses the criterion, but there are significant weaknesses.*

*3– **Good.** The proposal addresses the criterion well, but a number of shortcomings are present.*

*4– **Very good.** The proposal addresses the criterion very well, but a small number of shortcomings are present.*

*5– **Excellent.** The proposal successfully addresses all relevant aspects of the criterion.Any shortcomings are minor.*

Criterion 1 - Excellence

Score: **4.50** (Threshold: 3/5.00 , Weight: -)

The following aspects will be taken into account, to the extent that the proposed work corresponds to the topic description in the work programme:

Clarity and pertinence of the objectives

A brief introduction excellently identifies a topic of the proposed research: homelessness is an increasing social fact, and demonstrates its relevance to the scope of the Call. At the same time, the focus on long-term homelessness in Europe as a case study on inequality in 9 EU Member States provides a relatively limited potential for comprehensive analysis on inequalities in general.

The objectives of the projects are well-explained and they are in line with the second dimension of the challenge.

The relation to the work programme is structured in the table and each of the consortium response is very well described in regard to the topic of the proposal (long-term homelessness). The responses constitute the overview of the research programme and demonstrate its credibility very well.

Soundness of the concept, and credibility of the proposed methodology

The concept of the project is solid and soundly formulated in the proposal. The proposed methodology is convincingly explained and it is credible. The conceptual framework consists of three approaches, including an ecological perspective, which are very well explained. The overall method of the empirical framework is well specified in the comprehensive table. Incorporation of a gender perspective in methodology is foreseen and convincingly discussed.

Extent that proposed work is beyond the state of the art, and demonstrates innovation potential (e.g. ground-breaking objectives, novel concepts and approaches, new products, services or business and organisational models)

The proposed work is beyond the state of the art and the proposal demonstrates a substantial innovation potential. The innovation potential is represented by the scope of the study, from the individual-based treatment to the ecologic approach, which intends to focus on social environment. The consortium participants propose a novel approach, will generate new knowledge, propose novel policies and draft guidelines for social services.

Appropriate consideration of interdisciplinary approaches and, where relevant, use of stakeholder knowledge

The proposal guarantees an appropriate interdisciplinary approach, as the consortium participants will gather data using measures developed in psychology, philosophy, economy and health. In addition, use of relevant stakeholder knowledge is foreseen.

Gender as a factor determining homelessness is adequately addressed in the proposal, with one team having specific expertise in this area. Research teams appropriately take the issue of gender balance into consideration and the participation of women to the research is guaranteed at all levels, including as work package leaders.

Criterion 2 - Impact

Score: **5.00** (Threshold: 3/5.00 , Weight: -)

The following aspects will be taken into account:

The extent to which the outputs of the project would contribute to each of the expected impacts mentioned in the work programme under the relevant topic

The main expected impacts are clearly identified and convincingly explained, although mainly in relation to homelessness. In addition, the original perspective how the challenges listed in the Call has the potential to advance both the theory and the practice of social justice, reverse inequalities and unfairness is structured and explained in the comprehensive table.

The potential obstacles for the project in terms of agency are adequately identified and discussed.

Any substantial impacts not mentioned in the work programme, that would enhance innovation capacity, create new market opportunities, strengthen competitiveness and growth of companies, address issues related to climate change or the environment, or bring other important benefits for society

The proposal has the potential to contribute to the reduction of homelessness.

Quality of the proposed measures to:
- exploit and disseminate the project results (including management of IPR), and to manage research data where relevant
- communicate the project activities to different target audiences

The strategy of the exploitation and dissemination of the project results is well described. The consortium has built a network over several years, which help to the dissemination. Moreover, a European network focused on the same thematic will organize regular conferences.

The main target groups as well as the non-academic partners in exploitation and dissemination of the project results are specified.

The communication activities are planned with appropriate details, making use of 'traditional' (conferences, lectures, publications etc.) means, as well as digital technologies (website, facebook etc).

The data management plan including IPR and knowledge management is adequately outlined. The responsible bodies are identified: a communication team who give a one-month paper to the media, and help all partners to communicate.

Criterion 3 - Quality and efficiency of the implementation

Score: **5.00** (Threshold: 3/5.00 , Weight: -)

The following aspects will be taken into account:

Quality and effectiveness of the work plan, including extent to which the resources assigned to work packages are in line with their objectives and deliverables

The overall quality and effectiveness of the work plan is very good: it convincingly described and supported by the overview of the interrelated WPs as well. The resources assigned to the various work packages are appropriate. In addition, the extremely rich list of deliverables is

presented.

Appropriateness of the management structures and procedures, including risk and innovation management

The management structures and procedures, including decision making mechanisms and a strategy of conflict resolution and risks of implementation are convincingly described and they are appropriate the needs of the project; two WPs are dedicated to the management. Innovation management is adequately addressed. At the same time a graphical presentation of the management structures would facilitate perception of the management strategy.

Complementarity of the participants and extent to which the consortium as a whole brings together the necessary expertise

The Consortium as a whole is adequately described. The teams have relevant expertise in various areas of the social sciences and humanities and the consortium participants include partners with diversified and complementary expertise in the field of homelessness.

Appropriateness of the allocation of tasks, ensuring that all participants have a valid role and adequate resources in the project to fulfil that role

The resources to be committed are convincingly described, justified and appropriate the needs of the project: tasks are allocated in an appropriate way among consortium participants; roles are determined by the specific expertise of the team members and adequate resources are allocated.

Scope of the proposal

Status: **Yes**

Comments (in case the proposal is out of scope)

Not provided

Operational Capacity

Status: **Operational Capacity: Yes**

If No, please list the concerned partner(s), the reasons for the rejection, and the requested amount.

Not provided

Exceptional funding of third country participants/international organisations

A third country participant/international organisation not listed in General Annex A to the Main Work Programme may exceptionally receive funding if their participation is essential for carrying out the project (for instance due to outstanding expertise, access to unique know-how, access to research infrastructure, access to particular geographical environments, possibility to involve key partners in emerging markets, access to data, etc.). (For more information, see the Online Manual)

Based on the information provided in the proposal, I consider that the following participant(s)/international organisation(s) that requested funding should exceptionally be funded:
(Please list the Name and acronym of the applicant, Reasons for exceptional funding and the Requested grant amount.)

Not provided

Based on the information provided in the proposal, I consider that the following participant(s)/international organisation(s) that requested funding should NOT be funded:
(Please list the Name and acronym of the applicant, Reasons for exceptional funding and the Requested grant amount.)

Not provided

Use of human embryonic stem cells (hESC)

Status: **No**

If yes, please state whether the use of hESC is, or is not, in your opinion, necessary to achieve the scientific objectives of the proposal and the reasons why. Alternatively, please also state if it cannot be assessed whether the use of hESC is necessary or not because of a lack of information.

Not provided

Digitally sealed by the European Commission
Date: 2016.05.20 10:01:18 CEST

This document is digitally sealed. The digital sealing mechanism uniquely binds the document to the modules of the Participant Portal of the European Commission, to the transaction for which it was generated and ensures its integrity and authenticity.

Any attempt to modify the content will lead to a breach of the electronic seal, which can be verified at any time by clicking on the digital seal validation symbol.
